# Therapeutic enhancement of blood–brain and blood–tumor barriers permeability by laser interstitial thermal therapy

**DOI:** 10.1093/noajnl/vdaa071

**Published:** 2020-06-30

**Authors:** Afshin Salehi, Mounica R Paturu, Bhuvic Patel, Matthew D Cain, Tatenda Mahlokozera, Alicia B Yang, Tsen-Hsuan Lin, Eric C Leuthardt, Hiroko Yano, Sheng-Kwei Song, Robyn S Klein, Robert Schmidt, Albert H Kim

**Affiliations:** 1 Department of Neurological Surgery, Washington University School of Medicine in St. Louis, St. Louis, Missouri, USA; 2 Department of Medicine, Washington University School of Medicine in St. Louis, St. Louis, Missouri, USA; 3 Department of Radiology, Washington University School of Medicine in St. Louis, St. Louis, Missouri, USA; 4 Department of Neurology, Washington University School of Medicine in St. Louis, St. Louis, Missouri, USA; 5 Department of Genetics, Washington University School of Medicine in St. Louis, St. Louis, Missouri, USA; 6 Department of Neuroscience, Washington University School of Medicine in St. Louis, St. Louis, Missouri, USA; 7 Department of Pathology and Immunology, Washington University School of Medicine in St. Louis, St. Louis, Missouri, USA

**Keywords:** blood–brain barrier, blood–tumor barrier, central nervous system tumors, glioblastoma, laser interstitial thermal therapy

## Abstract

**Background:**

The blood–brain and blood–tumor barriers (BBB and BTB), which restrict the entry of most drugs into the brain and tumor, respectively, are a significant challenge in the treatment of glioblastoma. Laser interstitial thermal therapy (LITT) is a minimally invasive surgical technique increasingly used clinically for tumor cell ablation. Recent evidence suggests that LITT might locally disrupt BBB integrity, creating a potential therapeutic window of opportunity to deliver otherwise brain-impermeant agents.

**Methods:**

We established a LITT mouse model to test if laser therapy can increase BBB/BTB permeability in vivo. Mice underwent orthotopic glioblastoma tumor implantation followed by LITT in combination with BBB tracers or the anticancer drug doxorubicin. BBB/BTB permeability was measured using fluorimetry, microscopy, and immunofluorescence. An in vitro endothelial cell model was also used to corroborate findings.

**Results:**

LITT substantially disrupted the BBB and BTB locally, with increased permeability up to 30 days after the intervention. Remarkably, molecules as large as human immunoglobulin extravasated through blood vessels and permeated laser-treated brain tissue and tumors. Mechanistically, LITT decreased tight junction integrity and increased brain endothelial cell transcytosis. Treatment of mice bearing glioblastoma tumors with LITT and adjuvant doxorubicin, which is typically brain-impermeant, significantly increased animal survival.

**Conclusions:**

Together, these results suggest that LITT can locally disrupt the BBB and BTB, enabling the targeted delivery of systemic therapies, including, potentially, antibody-based agents.

**Key Points**

- Laser therapy increases BBB and BTB permeability up to 30 days posttreatment and enables brain entry of large-molecular-weight moieties.
- Laser therapy increases BBB and BTB permeability by disrupting endothelial cell tight junctions and increasing transcytosis.
- In a preclinical mouse model, laser ablation with adjuvant chemotherapy significantly improved animal survival.

**Importance of the Study**

The blood–brain and blood–tumor barriers (BBB and BTB), which restrict the entry of therapeutic drugs to the brain, are a significant challenge in the treatment of glioblastoma. We established a laser interstitial thermal therapy (LITT) mouse model and tested if LITT can increase BBB and BTB permeability. We demonstrated that LITT disrupts the BBB and BTB over a sustained duration and enables therapeutic agents and molecules as large as antibodies to enter the central nervous system (CNS). Furthermore, our preclinical mouse experiments provide proof of the concept that laser-induced BBB/BTB disruption combined with an anticancer drug can increase survival in glioblastoma tumor-bearing mice. These results have implications for the treatment of various CNS tumors.

Glioblastoma is the most common malignant primary brain tumor, which is characterized by an aggressive natural history and poor prognosis. Although advances have been made along multiple therapeutic fronts, median survival for this disease remains about 15 months, with a 5-year survival of less than 10%.^1^ Other than the standard-of-care drug, temozolomide, most systemic chemotherapeutics have been unsuccessful in large part from the blood–brain barrier (BBB) restricting the delivery of anticancer drugs to tumor-infiltrated brain.^2^ Blood–tumor barrier (BTB) permeability is more complex, as angiogenesis in glioblastoma leads to the formation of abnormal tumor microvessels, which exhibit limited and heterogeneous permeability.^3^ Additionally, established microvessels feeding the infiltrating edge of the tumor are as impermeable as the normal BBB.^4^ Thus, there is an urgent need to identify new approaches to bypass the BBB and BTB to deliver therapeutic drugs to glioblastomas and other diseases of the CNS.

Several strategies have been developed to increase delivery of therapeutic compounds to brain tumors, including modification of drug structure or use of carrier molecules, direct delivery to the tissue, chemical disruption of the BBB, and local physical disruption of the BBB.^5–7^ Local disruption of the BBB in regions of pathology carries certain advantages, including flexibility of combinations with different systemically delivered drugs while mitigating drug delivery and BBB leakiness in the unaffected brain. A recent study suggested that laser interstitial thermal therapy (LITT) could disrupt the local peritumoral BBB in tumor patients.^8^ LITT is a minimally invasive ablative technique that has shown promise in the treatment of brain tumors, including glioblastoma, other gliomas, and brain metastases.^9–11^ The tissue surrounding the laser absorbs emitted photons, causing controlled local hyperthermia and tumor cell death. At the periphery of the laser-treated zone, LITT increases contrast enhancement along with other MR markers of BBB disruption in human patients.^8^ However, there is little direct evidence for LITT-induced changes in BBB and BTB permeability, and fundamental characteristics of the LITT effect on BBB/BTB permeability such as molecular size threshold and mechanism of action remain to be explored.

To address these questions, we developed a mouse model of LITT and show that laser treatment increases BBB and BTB permeability over an extended period following treatment. We also identify mechanisms underlying these changes in BBB and BTB permeability. Finally, we take advantage of this property of laser treatment to augment local tumor access of an anticancer drug in an animal glioblastoma model. Taken together, this work provides the foundation for a novel laser-based approach to locally treating malignant tumors in the brain.

## Materials and Methods

### Cell Culture

Glioma 261 (GL261)^12^ cells (a generous gift from Dr Gavin Dunn, Washington University, St. Louis), bEnd.3 (ATCC CRL-2299), and 293LE cells were cultured in DMEM (Sigma) with 10% fetal bovine serum (Thermo-Fischer Scientific) and 1:100 penicillin/streptomycin. MGG8 human glioblastoma cells (a generous gift from Drs Hiroaki Wakimoto and Daniel Cahill, Harvard Medical School) were cultured as spheres in Neurobasal medium supplemented with N2, B27, and nonessential amino acids (Thermo-Fischer Scientific) plus EGF and bFGF (Peprotech, 20 ng/mL).^13^ All cells were incubated at 37°C with 5% (vol/vol) CO_2_. Viral transduction was performed as described.^14^

### Laser Interstitial Thermal Therapy

All animal experiments were in accordance with a protocol approved by the Animal Studies Committee at Washington University and compliant with the recommendations of the Guide for the Care and Use of Laboratory Animals (NIH). A stereotactic apparatus was used to target coordinates 0.25 mm rostral to bregma, 2 mm lateral, and 2 mm deep. Laser therapy was delivered using a Dornier Medilas Fibertom 8100 at 2 W power on continuous mode to deliver an Nd-YAG laser at 1064 nm through a 600 µm fiber optic cable. The temperature was measured with a 400 nm thermocouple 1 mm rostral to the laser fiber. The laser was manually switched on and off to maintain the desired temperature ranges at 43**°**C. Laser-treated mice were injected with 30 mg/kg of phenytoin intraperitoneally for seizure prophylaxis.

### Orthotopic Tumor Implantation

Tumor cell injections were performed as described.^15^ About 50 000 cells in 3 μL of Matrigel (Corning) or phosphate-buffered saline were stereotactically injected into the right somatosensory cortex of 6-week-old mice (C57BL/6J for GL261, NOD-SCIDγ for MGG8) at coordinates 0.25 mm rostral to bregma, 2 mm lateral, and 2 mm deep using a 10 µL Hamilton syringe. Tumors were allowed to grow for 7–10 days prior to subsequent experimentation (specified in Figure Legends). To monitor tumor burden, live bioluminescence imaging was performed as described.^15^

### In Vivo BBB and BTB Permeability Assays

Fluorescein permeability assay was performed as described.^16^ Briefly, mice were injected with an intraperitoneal dose of sodium fluorescein solution (Sigma-Aldrich). Mice were euthanized, and blood and brain tissue collected, with the harvesting of an approximately 3 mm × 3 mm × 3 mm cube of fluorescein-permeable and corresponding control brain area identified via monocular microscopy (Amscope). For dextran/IgG permeability, 10 kDa and 70 kDa dextran (Thermo-Fischer Scientific) or human IgG (Sigma-Aldrich) were injected intravenously (retro-orbital) in mice (see Supplementary Materials).

### Histopathology and Immunofluorescence

For histology, brain sections were stained with hematoxylin and eosin. For immunofluorescence, frozen sections or confluent bEnd.3 cells were stained with primary antibodies followed by incubation with corresponding Alexa fluorophore-conjugated secondary antibodies (Thermo-Fisher Scientific). Immunofluorescence images were analyzed via corrected total fluorescence on ImageJ (NIH; see Supplementary Materials).

### Doxorubicin Permeability and Quantification

Briefly, doxorubicin (Sigma-Aldrich, 16 mg/kg in sterile water) was injected intravenously into mice. Mice were anesthetized and perfused and treated brain regions harvested. Brain homogenates were used to measure doxorubicin concentration by fluorometry.^17^ For bioluminescence live imaging (BLI) and survival experiments, 12 mg/kg of doxorubicin was used for the first dose and 8 mg/kg of doxorubicin for the second dose (see Supplementary Materials).

### Statistics

All experiments including images are representative of results from 3 independent experiments unless otherwise stated and were quantified in a blinded fashion. In animal experiments, mice were randomized to different treatment arms. Statistical analyses were performed with GraphPad Prism 8.0.2. The unpaired Student’s *t*-test was used for comparisons in experiments with only 2 groups. In experiments with more than 2 comparison groups, ANOVA was performed with Bonferroni post-hoc test for pairwise comparisons. *P* < .05 was considered significant.

## Results

### Establishing a Mouse Model of LITT

**Figure 1.**
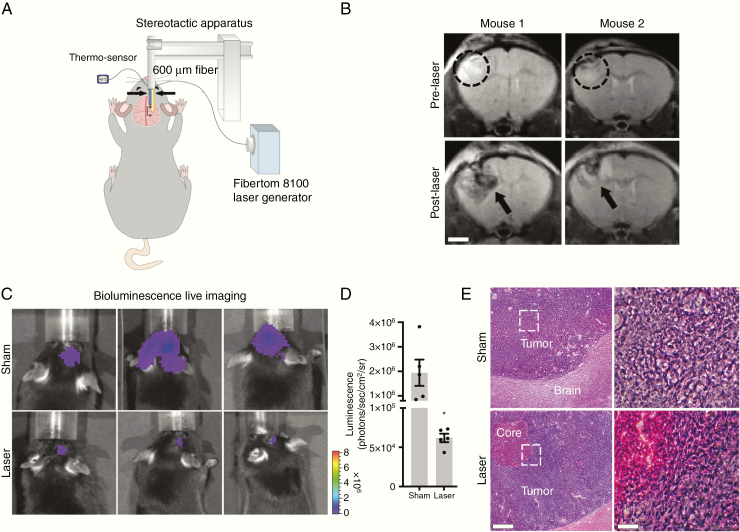
Establishment of a LITT mouse model. (A) Schematic depiction of the LITT delivery system in mice. The laser fiber (right arrow) is positioned 1 mm caudal to the thermo-sensor (left arrow). (B) Animals stereotactically implanted with GL261 tumor cells were subjected to MRI 7 days later. Representative T2-weighted MR images of 2 mice before and 24 h after LITT are shown. Tumor (dashed circle) and LITT-treated area (black arrow) are highlighted (*n* = 3 for each). Scale bar = 2 mm. (C) Animals stereotactically implanted with luciferase-expressing GL261 tumor cells were treated 8 days later with LITT or sham. (D) Tumor volume was quantified by BLI 3 days posttreatment. LITT-treated animals had significantly lower tumor burden compared to sham (*n* = 5 for each condition, unpaired *t*-test, *P < .01). (E) Representative H&E stained sections of sham (top) and laser-treated (bottom) mouse brains are shown (*n* = 3 for each condition). Loss of nuclear hematoxylin staining and enhanced eosin staining are observed in the necrotic laser core. Scale bar = 500 µm, 100 µm.

We established a mouse model to stereotactically deliver laser treatment into either the mouse somatosensory cortex or an orthotopically implanted brain tumor (Figure 1A). To model glioblastoma, GL261 cells were stereotactically injected into the somatosensory cortex of C57BL/6J mice and then treated with LITT 7–10 days later (Figure 1B–E). Laser treatment was delivered for up to 3 min while a co-inserted thermocouple sensor 1 mm from the laser fiber was used to maintain tissue temperatures at least 43**°**C (Supplementary Figure S1) to model laser therapy delivered in humans. Temperatures at the laser-treated core of the tumor reached more than 50**°**C, resulting in irreversible cell death (Figure 1E; Supp1ementary Figures S1 and S2). Magnetic resonance imaging (MRI) was performed pre- and post-LITT on tumor-bearing mice, which demonstrated reproducible targeting of brain tumors (Figure 1B). Post-LITT MRI of tumor-bearing mice showed a central area of heterogeneous T2W hypointensity, consistent with coagulative necrosis and blood products as well as a halo of T2W hyperintensity, indicating edema (Figure 1B), similar to the imaging characteristics described in human LITT.^18^ To demonstrate that LITT can ablate tumor cells in vivo, we stereotactically injected luciferase-expressing GL261 intracranially in mice to monitor tumor burden by BLI. Tumor burden was significantly lower in laser- versus sham-treated mice 3 days after treatment (Figure 1C and D). Accordingly, histopathological analysis of laser-treated tumors showed loss of nuclei and increased eosin staining in the laser core, consistent with tumor cell necrosis.^19^ Transmission electron microscopy of the native brain treated with LITT showed similar results. Three days after laser treatment, we observed widespread necrotic tissue injury, loss of cellular adhesion, and the presence of red blood cells from vessel destruction in the core. In a concentric area of the brain adjacent to and outside of the necrotic laser core, we observed relatively preserved blood vessels and normal surrounding neuropil (Supplementary Figure S3).

### BBB and BTB Permeability Are Increased by Laser Treatment

**Figure 2.**
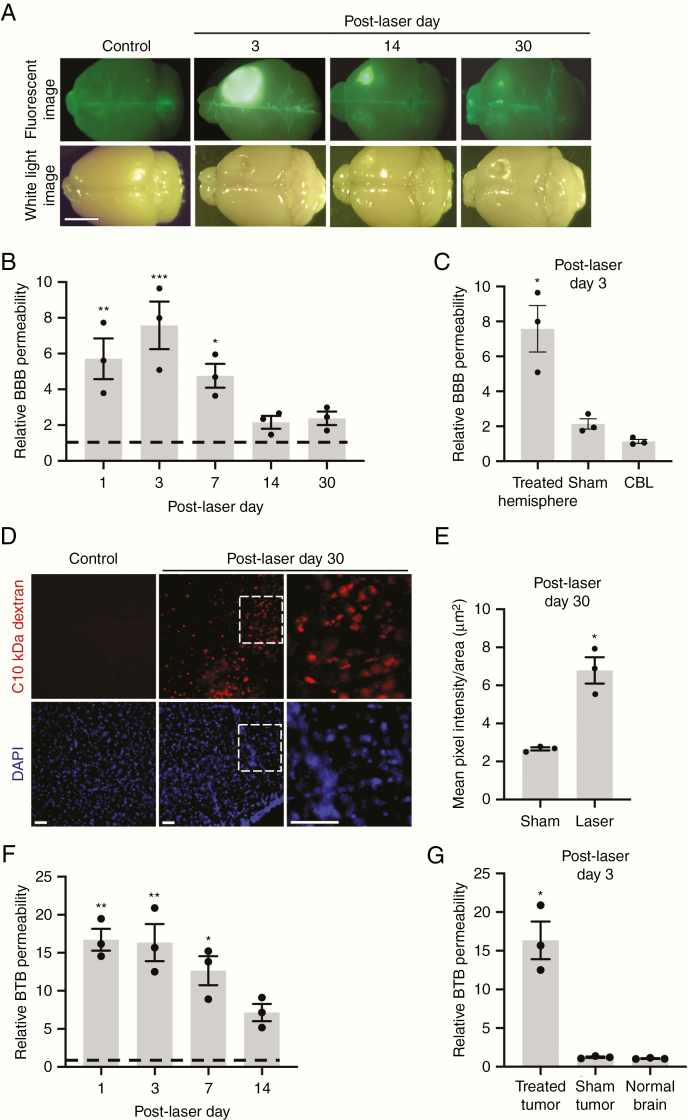
LITT increases BBB and BTB permeability in vivo. (A) Representative white light and fluorescence images of mouse brains harvested on the indicated days after intravenous fluorescein injection (*n* = 3 for each condition). LITT was performed in the right somatosensory cortex. Control = unmanipulated brain. Scale bar = 5 mm. (B) Animals treated as in A were assessed for tissue fluorescein uptake (normalized to control brains) on indicated post-LITT days. Control = 1 (dotted line). Data represent mean ± SEM. Laser increased BBB permeability compared to control (*n* = 3 for each condition, ANOVA, *P < .05, **P < .01, ***P < .001). (C) Relative BBB permeability was measured as in B on post-laser day 3 in indicated brain regions. LITT increased BBB permeability in the treated forebrain hemisphere, but not in the cerebellum (CBL) of the same brain or sham treatment (insertion of optical fiber) (*n* = 3 for each condition, ANOVA, *P < .01). (D) Animals treated with laser were injected intravenously with 10 kDa dextran on post-LITT day 30, and brains processed for immunofluorescence on free-floating 50 µm sections to assess dextran penetration. Representative images are shown (*n* = 3 for each condition). Scale bar = 50 µm. (E) Brain permeability of 10 kDa dextran was quantified by the mean pixel intensity of fluorescence divided by area (µm^2^). Data represent mean ± SEM (*n* = 3 for each condition, unpaired *t*-test, *P < .01). (F) Animals were orthotopically implanted with GL261 tumor cells, and laser treatment to the tumor performed 7 days later. Relative BTB permeability (normalized to control brain) was assessed by quantification of tissue fluorescein uptake on indicated post-LITT days. Control = 1 (dotted line). LITT increased BTB permeability compared to control (*n* = 3 for each condition, ANOVA, *P < .01, **P < .001). (G) Relative BTB permeability was measured as in (F) on post-laser day 3 following LITT (treated) or sham treatment. The normal brain represents a contralateral forebrain hemisphere with no tumor and not treated with laser. Laser treatment increased BBB permeability in the tumor-bearing hemisphere compared to sham treatment and contralateral hemisphere (*n* = 3 for each condition, ANOVA, *P < .001).

To determine if LITT directly affects BBB permeability in mice, we intravenously injected fluorescein at several time points following laser or sham treatment in native brain tissue and then harvested treated brain areas for analysis (Figure 2A). Relative BBB permeability, as measured by brain fluorescein accumulation, substantially increased within the first week post-LITT before gradually decreasing (Figure 2B). LITT-treated hemispheres demonstrated a significantly greater degree of fluorescein permeability compared to sham-treated hemispheres and the cerebellum, a distant site in LITT-treated mice, indicating treatment and location-specific BBB effect (Figure 2C). Although fluorescein accumulation in laser-treated brains was visibly evident by fluorescence imaging on post-LITT days 14 and 30, the increase in quantitative fluorescein tracer within the hemisphere was not significant at these time points, likely due to decreased assay sensitivity for detecting more minute differences in fluorescein accumulation (Figure 2A and B). We therefore adopted a complementary, more sensitive, immunofluorescence-based approach to determine if laser-induced BBB permeability extends beyond the 7 days identified by the fluorescein assay. Using intravenous 10 kDa dextran as a second tracer for BBB integrity, we observed a significant increase in BBB permeability on post-LITT day 30 (Figure 2D and E).

Similarly, to determine the integrity of the BTB, quantitative fluorescein permeability assays were performed in mice bearing GL261 tumors following LITT to the tumor (Figure 2F and G). Laser treatment of tumors significantly increased fluorescein tracer infiltration in tumor-bearing hemispheres compared to that of untreated control tumors for at least 2 weeks post-LITT (Figure 2F). Laser-treated tumors demonstrated a significantly greater degree of fluorescein permeability compared to sham-treated tumors and normal control brains (Figure 2G).

### LITT Increases BBB and BTB Permeability to Large Molecules

**Figure 3.**
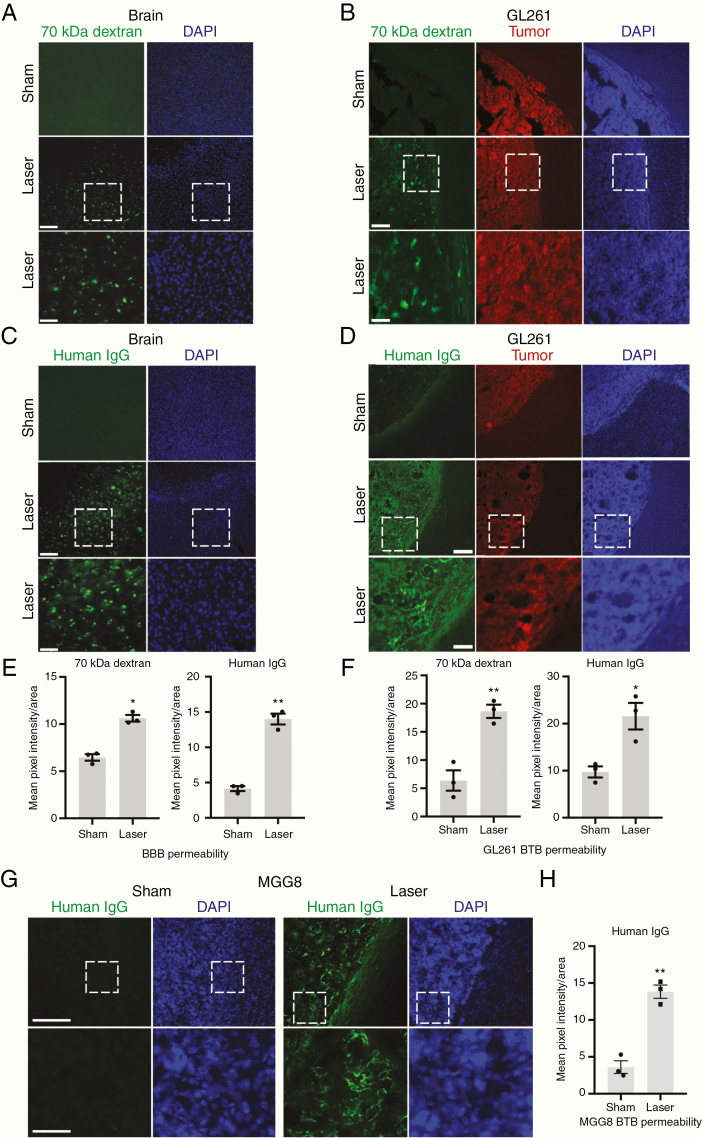
LITT increases BBB and BTB permeability to large molecules. (A) Animals treated with LITT were injected intravenously with 70 kDa dextran on post-laser day 3, and brains harvested and processed for immunofluorescence to assess dextran penetration. Representative images are shown (*n* = 3 for each condition). Scale bar = 100 µm; scale bar for magnified images = 300 µm. (B) Mice with stereotactically injected RFP-expressing GL261 cells were treated with LITT 10 days after implantation and injected intravenously with 70 kDa dextran on post-LITT day 3. Brains were processed as in A. Representative images are shown (*n* = 3 for each condition). (C) Mice treated with LITT as in A were injected intravenously with human IgG on post-laser day 3, and brains processed to assess IgG tissue penetration. Representative images are shown (*n* = 3 for each condition). (D) Tumor-bearing mice as in B were treated with LITT 10 days after implantation and injected intravenously with human IgG on post-LITT day 3. Brains were processed as in C. Representative images are shown (*n* = 3 for each condition). (E) Brain permeability of 70 kDa dextran and human IgG in LITT- and sham-treated brain was quantified by mean pixel intensity of fluorescence divided by area (µm^2^). Data represent mean ± SEM. LITT significantly increased penetration of 70 kDa dextran and human IgG compared to sham (*n* = 3 for each condition, *t*-test, *P < .01, **P < .001). (F) Brain tumor permeability of 70 kDa dextran and human IgG in laser- and sham-treated brain was quantified by mean pixel intensity of fluorescence divided by area (µm^2^). Data represent mean ± SEM. Laser treatment significantly increased tumor penetration of 70 kDa dextran and human IgG (*n* = 3 for each condition, *t*-test, *P < .05, **P < .01). (G) NOD-SCIDγ mice were stereotactically injected with MGG8 human glioblastoma cells and subjected to sham or laser treatment 10 days after implantation. Animals were injected intravenously with human IgG on post-laser day 3, and brains processed with immunofluorescence to assess IgG tissue penetration. Representative images are shown (*n* = 2 for each condition). DAPI = nuclear stain. Scale bar = 100 μm. Magnified image scale bar = 400 μm. (H) Tissue was processed as in D and BTB permeability quantified as in F for MGG8 tumor-bearing mice. Data represent mean ± SEM. Laser treatment significantly increased tumor penetration of human IgG (*n* = 3 for each condition, *t*-test, **P < .002).

Our results demonstrate that local BBB and BTB integrity is decreased in LITT-treated brain and brain tumors, raising the possibility that larger molecular weight moieties might also be capable of permeating the BBB and BTB following LITT. We therefore intravenously injected 70 kDa dextran in mice 3 days following laser or sham treatment (Figure 3A). As a positive control, an intravenous injection was confirmed by the detection of dextran in the liver (Supplementary Figure S4). We observed increased 70 kDa dextran infiltration particularly outside of the laser core, in the *laser penumbra*, a concentric region adjacent to and surrounding the core, which was not evident in the sham-treated brain (Figure 3A). Quantification of dextran fluorescence intensity in brain parenchyma indicated a significant increase in laser-induced BBB permeability (Figure 3E). We then investigated whether laser-induced permeability could allow even larger molecules, such as antibodies, to penetrate the BBB. Human IgG, which is approximately 150 kDa, was intravenously injected in mice 3 days after laser treatment. Immunostaining of human IgG in laser-treated mouse brain also demonstrated widespread infiltration predominantly in the laser penumbra with little IgG infiltration in sham-treated brain (Figure 3C and E). In parallel, we tested if LITT increases BTB permeability to large molecules. Analysis of LITT-treated GL261 tumor-bearing mice demonstrated increased 70 kDa dextran and human IgG permeability in brain tumors (Figure 3B, D, and F). As with laser-treated brain, an inspection of the spatial pattern of laser-induced BTB permeability revealed little tumor penetration of both large-molecular-weight tracers in the necrotic core of laser-treated tumors and extensive infiltration in the surrounding laser penumbra within tumor regions (Supplementary Figure S5).

We confirmed that LITT increases BTB permeability in an additional mouse model of glioblastoma, using a human patient-derived glioblastoma sphere line (MGG8) implanted intracranially in NOD-SCIDγ mice (Figure 3G).^13,20^ Quantification of human IgG fluorescence intensity in brain parenchyma indicated a significant increase in laser-induced BTB permeability (Figure 3H). To verify that intravenous human IgG penetrates LITT-treated tumor parenchyma and does not merely reside in blood vessels, we co-stained for human IgG and the endothelial marker CD31 in LITT-treated tumors. Substantial extravasation of IgG molecules was observed outside of blood vessels in LITT-treated tumors compared to that of sham treatment (Supplementary Figure S6A). Quantification of the IgG permeability index indicated a significant increase in the delivery of IgG to tumor parenchyma outside of blood vessels following laser treatment (Supplementary Figure S6B). Together, these observations indicate that LITT can increase BBB and BTB permeability to small- and large-molecular-weight moieties predominantly in tissue regions surrounding the laser core.

### LITT Disrupts Tight Junction Integrity In Vivo and In Vitro

**Figure 4.**
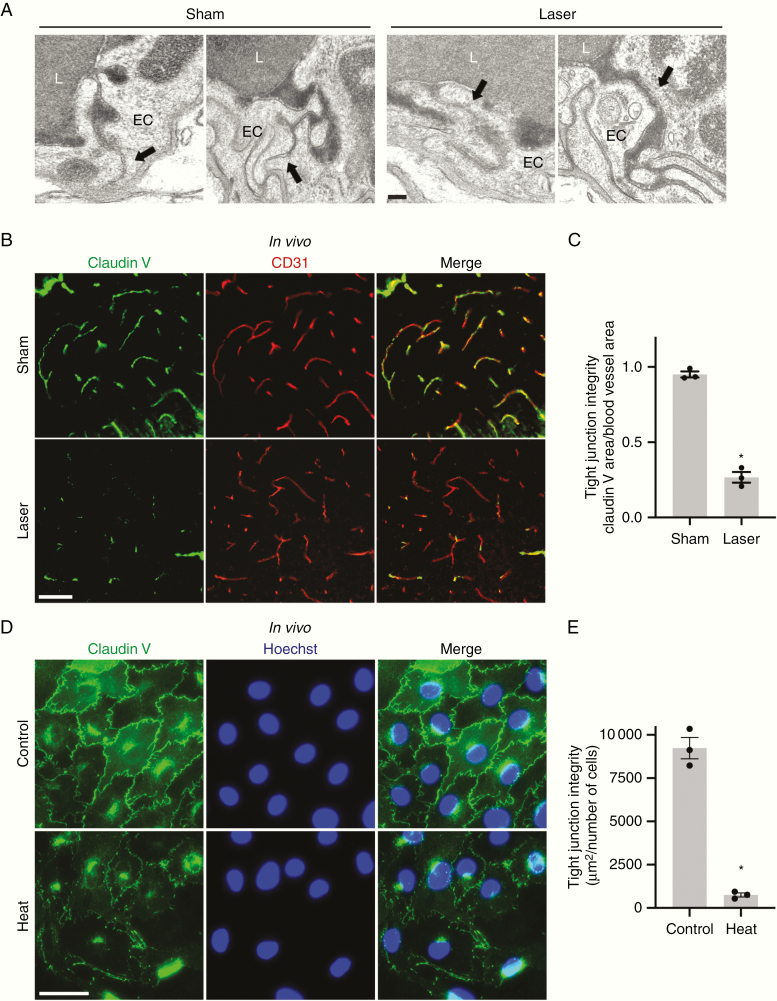
LITT disrupts brain endothelial cell tight junctions. (A) Representative transmission electron microscopy images are shown following intravenous HRP injection in sham and laser-treated brains (*n* = 3 for each condition). Intact tight junctions (arrows) are observed in the control brain whereas tight junction aberrancies (arrows) are seen in the laser penumbra of laser-treated brain. L, blood vessel lumen; EC, endothelial cell. Scale bar = 100 nm. (B) Brains of animals treated with LITT or sham treatment were processed on post-LITT day 3 for claudin V immunofluorescence. CD31, endothelial cell marker. Representative images are shown (*n* = 3 for each condition). Scale bar = 50 µm. (C) The integrity of brain endothelial cell tight junctions in mice treated as in B was quantified by area of claudin V immunostaining divided by the area of blood vessels (CD31 immunostaining, µm^2^). Data represent mean ± SEM. LITT significantly decreased tight junction integrity compared to sham (*n* = 3 for each condition, *t*-test, *P < .0001). (D) Brain endothelial cells (bEnd.3) were cultured, and claudin V immunofluorescence performed after exposure to heat (43°C) for 90 min. Representative images are shown (*n* = 3 for each condition). Scale bar = 50 µm. (E) The integrity of tight junctions in brain endothelial cells treated as in D was quantified by area of claudin V immunostaining divided by cell count. Data represent mean ± SEM. Heat significantly decreased tight junction integrity compared to control (*n* = 3 for each condition, *t*-test, *P < .01).

We next focused on the laser penumbra, which appeared not to harbor obvious cellular disruption by histology (Figure 1E; Supplementary Figure S3) but demonstrated tissue infiltration of BBB permeability tracers (Figure 3), to understand the mechanisms by which LITT increases BBB permeability. First, we investigated if LITT disrupts the functional integrity of brain endothelial cell tight junctions in vivo, an important mechanism of BBB maintenance.^21^ Mice treated with LITT were intravenously injected with horseradish peroxidase (HRP), and electron microscopy in the laser penumbra was performed to evaluate tight junction integrity (Figure 4A). Whereas sham-treated brains demonstrated intact tight junctions that halted luminal HRP at tight junction sealing strands, LITT-treated brains showed tight junction aberrancies with separated sealing strands and leakage of HRP toward the abluminal side in the laser penumbra (Figure 4A). We hypothesized that LITT might perturb the tight junction structure by altering the expression of key tight junction proteins. Brain endothelial cells express claudin V, a transmembrane protein concentrated in tight junctions and whose depletion is associated with BBB disruption in mice in vivo and human endothelial cells in vitro.^22^ Laser treatment of the brain triggered a more punctate pattern and a decrease in overall intensity of claudin V staining in endothelial cells of the laser penumbra (Figure 4B and C). To test if thermal treatment directly causes a decrease in claudin V protein, human endothelial cells (bEnd.3) cultured in vitro were heated to 43°C for 90 min followed by incubation at 37°C (Figure 4D and E). One day after thermal treatment, bEnd.3 cells showed a dramatic decrease in claudin V protein levels at the plasma membrane (Figure 4D and E) with little effect on cell viability (data not shown), suggesting a direct effect of thermal energy on tight junction dysfunction.

### LITT Promotes Endothelial Cell Transcytosis

**Figure 5.**
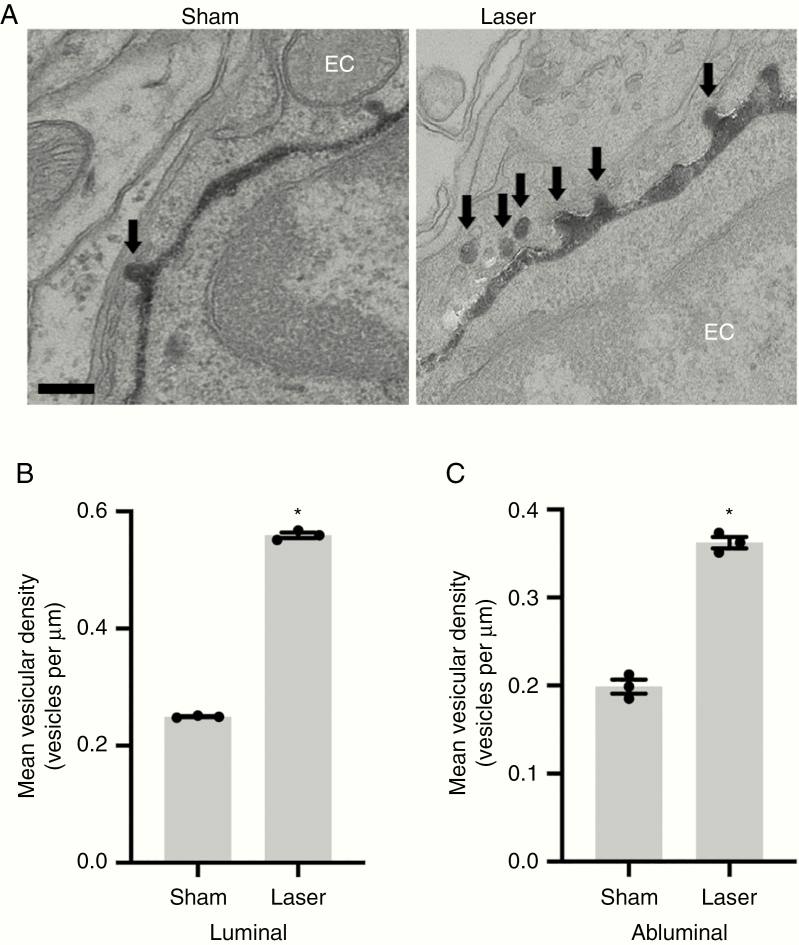
LITT increases endothelial cell transcytosis. (a) Representative transmission electron microscopy images are shown following intravenous HRP injection as in Figure 4A in sham and laser-treated brain on post-laser day 3 (*n* = 3 for each condition). HRP-filled vesicles within endothelial cells are highlighted (arrows). Scale bar = 100 nm. (B and C) Data represent mean ± SEM. Quantification of vesicular density (vesicles per μm) per length of luminal (B) and abluminal (C) membrane shows increased transcytosis in brain endothelial cells of laser-treated brain compared to sham-treated brain (*n* = 3 for each condition, 20 capillaries per animal, *t*-test, *P < .0001).

Recent reports indicate that active inhibition of endothelial cell transcytosis is another means of restricting BBB permeability under physiological conditions and can become dysregulated after pathological insults.^23,24^ Mice treated with LITT were intravenously injected with HRP, and brains harvested for electron microscopic analysis of HRP-containing vesicles in brain endothelial cells (Figure 5). Consistent with previous reports, endothelial cells in sham-treated brain demonstrated a low basal rate of transcytosis (Figure 5A).^25^ Strikingly, brain endothelial cells in the laser penumbra in LITT-treated mice demonstrated an increase in HRP-filled endocytic vesicles (Figure 5A). Mean vesicular density in endothelial cells within the laser penumbra was increased 2- to 3-fold compared to sham treatment (Figure 5C), suggesting endothelial cell transcytosis also contributes to the laser-induced elevation of BBB permeability.

### Laser-Mediated Drug Delivery to the Brain Increases Animal Survival

**Figure 6.**
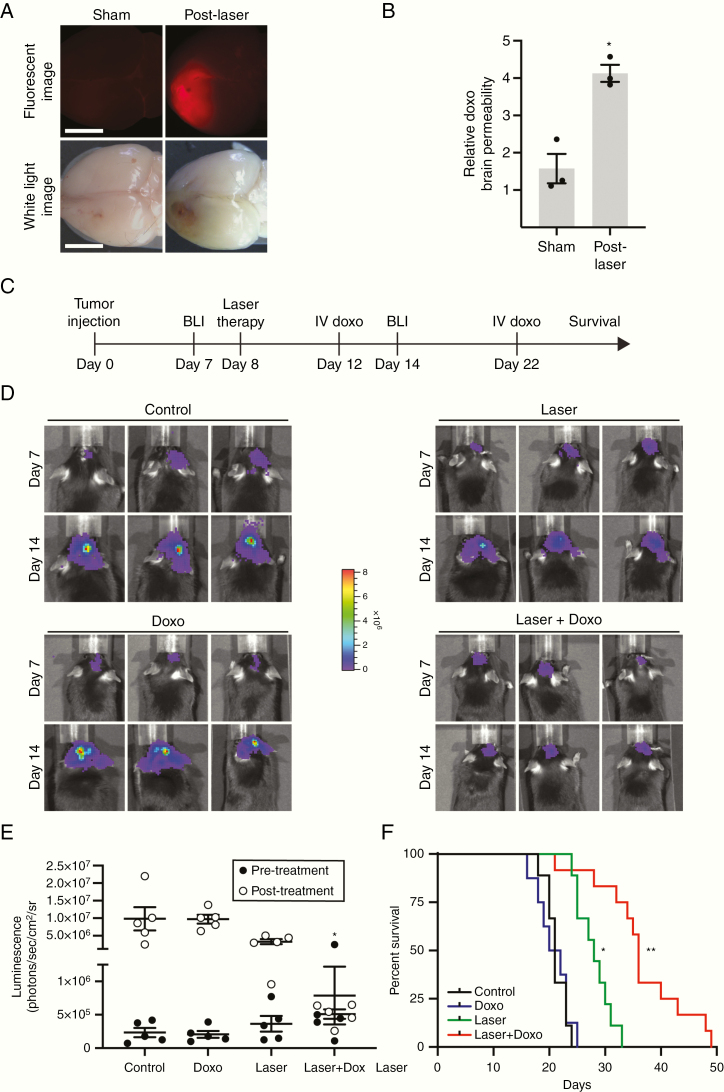
LITT enhances therapeutic drug delivery to the brain. (A) Mice were subjected to sham or laser treatment and were injected intravenously with doxorubicin (doxo) (16 mg/kg) on post-laser day 3. Three hours later, brains were harvested and imaged via white light or fluorescence for doxo signal. (B) Brains from mice treated as in A were harvested, and brain homogenates were quantified for doxo penetration via fluorimeter. Data represent mean ± SEM. Laser treatment significantly increased parenchymal doxo compared to sham (*n* = 3 for each condition, *t*-test, *P < .01). (C) Timeline of in vivo preclinical study to test LITT plus adjuvant doxo. (D) After orthotopic implantation of luciferase-expressing GL261 cells, BLI was performed on mice before a laser or sham treatment (day 7) and after indicated treatments in the same mice (day 14). Representative BLI images are shown from 3 animals in each group (*n* = 5 for each condition). Control = sham treatment plus intravenous vehicle; doxo alone = sham treatment plus intravenous doxo; laser alone = laser treatment plus intravenous vehicle. (E) Quantification of BLI images from animals treated as in D is shown. Laser plus doxo caused a significant decrease in tumor burden compared to control or doxo alone (*n* = 5 in each condition, ANOVA, *P < .05). (F) Mice treated as in D were monitored for neurological deficit-free survival (*n* = 8–12 for each condition). Whereas laser treatment alone showed a modest survival benefit compared to control or doxo (log-rank test with Bonferroni, *P < .0001), laser plus doxorubicin (2 doses, 12 mg/kg on day 4 and 8 mg/kg on day 14 post-laser) substantially increased survival compared to control, doxo, or laser alone (log-rank test with Bonferroni, **P < .0001, *P* < .0001, *P* < .004, respectively).

Having established that LITT increases local BBB/BTB permeability, we hypothesized that laser treatment might facilitate local drug delivery to brain tumors for therapeutic benefit. We tested if laser treatment could increase brain penetration of the chemotherapeutic agent doxorubicin because (1) doxorubicin is an effective anti-glioblastoma agent in vitro^26^ but is known to be BBB impermeant^27^ and (2) a current clinical trial is testing the efficacy of LITT plus doxorubicin for recurrent glioblastoma (clinicaltrials.gov, NCT01851733). Mice injected with GL261 tumors were treated with laser or sham treatment after 8 days and then intravenously injected with doxorubicin 3 days after probe insertion. Brains were assessed for doxorubicin penetration using a fluorescence-based assay (Figure 6A and B). Whereas sham-treated mice showed little evidence of parenchymal doxorubicin infiltration, LITT-treated mice showed substantial accumulation of brain tissue doxorubicin, indicating that laser treatment increases local BBB permeability to enable doxorubicin entry (Figure 6A and B).

We thus tested the hypothesis that LITT in combination with doxorubicin is therapeutically superior to either intervention alone in a preclinical model of glioblastoma (Figure 6C). C57BL/6J mice were intracranially injected with luciferase-expressing GL261 cells. After 8 days, animals were treated with laser or sham surgery to the tumor followed by dosing with intravenous doxorubicin or vehicle. BLI was performed to monitor tumor burden on all animals before and after treatments to ensure approximately equivalent initial tumor burden and to monitor subsequent therapeutic effects (Figure 6D and E). Although doxorubicin alone had no effect on tumor progression in mice, the combination of LITT plus doxorubicin significantly decreased tumor progression compared to control or doxorubicin alone (Figure 6E). A larger cohort of brain tumor-bearing animals treated in a similar manner were monitored for neurological deficit-free survival. Whereas tumor-bearing mice treated with laser demonstrated increased survival compared to control or doxorubicin-treated mice, mice treated with LITT plus doxorubicin showed significantly prolonged survival compared to mice in all other conditions including laser alone (Figure 6F). LITT plus doxorubicin (median survival 36 days) increased median survival by 71% compared to control treatment (median survival 21 days).

## Discussion

There is accumulating interest in LITT to treat brain pathologies due to its minimally invasive approach and associated faster recovery.^9–11^ Despite its increased clinical use over the past decade, there is little understanding of the biological effects of LITT on both tumor and the surrounding brain. To address this, we developed a mouse model of LITT. Our data show that LITT enables local delivery of small- and large-molecular-weight agents through the BBB and BTB over an extended period. While early experimental studies in animal models explored the general histopathological impact of LITT on normal brain and brain tumors, there has been little investigation of fundamental characteristics of the LITT effect on BBB/BTB permeability, such as molecular size threshold, duration, mechanism of action, and therapeutic efficacy.^28–31^ Our in vivo results provide direct evidence that the thermal effect of LITT disrupts the BBB and BTB at a targeted location. We demonstrate that laser treatment can greatly increase the amount of IgG that permeates laser-treated brain and brain tumors, raising the possibility that LITT may increase the delivery, and therefore efficacy, of therapeutic antibodies currently being used in clinical trials.^32,33^

We also sought to identify the mechanisms by which LITT alters BBB permeability. We discovered that at least one mechanism of laser-enhanced BBB permeability in vivo and in vitro occurs via tight junction disruption. Earlier studies indicated that whole-body hyperthermia (>40.0°C) alters glial and brain endothelial cell morphology.^34^ These studies, in conjunction with our in vitro experiments (Figure 4D and E), suggest the BBB effects of LITT operate through direct thermal effects on the endothelium. We also observed that, whereas control brain endothelial cells exhibit extremely low steady-state levels of transcytotic vesicles, laser-treated brain demonstrated an increased number of endothelial cell vesicles, suggesting that LITT increases the rate of transcytosis (Figure 5). The mechanisms by which endothelial cell transcytosis is inhibited in the brain are only beginning to become known.^24^ It will be of interest in future studies to determine if the endocytic vesicles observed following laser treatment represent macropinocytosis, clathrin-dependent endocytosis, or caveolae-mediated endocytosis.^35–37^ Gu et al.^25,38^ recently reported that vesicle formation and transcytosis are actively suppressed in brain endothelial cells via a Mfsd2a and caveolin-1-dependent mechanism. Although highly speculative, these findings raise the intriguing hypothesis that thermal energy may dysregulate Mfsd2a-dependent processes to increase endothelial cell transcytosis. Furthermore, it remains to be determined if secondary effects of LITT, such as the elaboration of cytokines or other inflammatory mediators, might also contribute to both LITT-induced transcytosis and/or endothelial tight junction disruption.^39^

This study has several limitations, primarily inherent to the use of a mouse animal model. Mice have different immunology, physiology, and potentially treatment response compared to humans. Other limitations include use of a thermocouple device, which provides temperature data at only specific points around the laser (core and 1 mm away), instead of MR thermometry (although MR thermometry is an indirect, calculated measure of temperature and is therefore not as accurate as the direct temperature measurements used in this study). There are also limitations associated with the tumor models used. GL261 elicits a more robust immune reaction in mice than GBM does in humans, and patient-derived xenografts (MGG8 line, Figure 3G) are limited by the immunocompromised setting in which they are implanted.

Our results suggest that combining transient BBB disruption via LITT with adjuvant antibody, biologic, or other pharmaceutical interventions may be considered for the treatment of glioblastoma and raises the intriguing possibility that LITT may increase access of drugs to additional brain pathologies. Further understanding of the specific mechanisms by which LITT causes changes in BBB and BTB properties is needed, as such information will provide insight into how to optimize the timing of drug delivery after LITT and, potentially, to augment LITT-induced BBB effects.

## Funding

This work was supported by the National Institutes of Health (R01NS094670 to A.H.K., R25NS090978 to A.S., R25NS090978 to B.P., R01NS052632 to R.S.K., P50/CA09056 to the Molecular Imaging Center at Washington University); Monteris Medical research grant (to A.H.K.); the Christopher Davidson and Knight Family Fund (to A.H.K.); the Duesenberg Research Fund (to A.H.K.); HDTRA 1-15-1-0032 (to R.S.K.).

## Supplementary Material

vdaa071_suppl_Supplementary_Figure_1

vdaa071_suppl_Supplementary_Figure_2

vdaa071_suppl_Supplementary_Figure_3

vdaa071_suppl_Supplementary_Figure_4

vdaa071_suppl_Supplementary_Figure_5

vdaa071_suppl_Supplementary_Figure_6

vdaa071_suppl_Supplementary_Figure_Legends

vdaa071_suppl_Supplementary_Material

## References

[1] Wen PY, Kesari S Malignant gliomas in adults. N Engl J Med. 2008;359(5):492–507. 18669428 10.1056/NEJMra0708126

[2] Langen UH, Ayloo S, Gu C Development and cell biology of the blood-brain barrier. Annu Rev Cell Dev Biol. 2019;35:591–613. 31299172 10.1146/annurev-cellbio-100617-062608 PMC8934576

[3] Thomsen MS, Routhe LJ, Moos T The vascular basement membrane in the healthy and pathological brain. J Cereb Blood Flow Metab. 2017;37(10):3300–3317. 28753105 10.1177/0271678X17722436 PMC5624399

[4] Zhao X, Chen R, Liu M, Feng J, Chen J, Hu K Remodeling the blood-brain barrier microenvironment by natural products for brain tumor therapy. Acta Pharm Sin B. 2017;7(5):541–553. 28924548 10.1016/j.apsb.2017.07.002 PMC5595291

[5] Lu CT, Zhao YZ, Wong HL, Cai J, Peng L, Tian XQ Current approaches to enhance CNS delivery of drugs across the brain barriers. Int J Nanomedicine. 2014;9:2241–2257. 24872687 10.2147/IJN.S61288 PMC4026551

[6] Zheng X, Shao X, Zhang C, et al. Intranasal H102 peptide-loaded liposomes for brain delivery to treat Alzheimer’s disease. Pharm Res. 2015;32(12):3837–3849. 26113236 10.1007/s11095-015-1744-9

[7] Poon C, McMahon D, Hynynen K Noninvasive and targeted delivery of therapeutics to the brain using focused ultrasound. Neuropharmacology. 2017;120:20–37. 26907805 10.1016/j.neuropharm.2016.02.014 PMC5028296

[8] Leuthardt EC, Duan C, Kim MJ, et al. Hyperthermic laser ablation of recurrent glioblastoma leads to temporary disruption of the peritumoral blood brain barrier. PLoS One. 2016;11(2):e0148613. 26910903 10.1371/journal.pone.0148613 PMC4766093

[9] Kamath AA, Friedman DD, Akbari SHA, et al. Glioblastoma treated with magnetic resonance imaging-guided laser interstitial thermal therapy: safety, efficacy, and outcomes. Neurosurgery. 2019;84(4):836–843. 30137606 10.1093/neuros/nyy375 PMC6425465

[10] Ahluwalia M, Barnett GH, Deng D, et al. Laser ablation after stereotactic radiosurgery: a multicenter prospective study in patients with metastatic brain tumors and radiation necrosis. J Neurosurg. 2018;130(3):804–811. 29726782 10.3171/2017.11.JNS171273

[11] Salehi A, Kamath AA, Leuthardt EC, Kim AH Management of intracranial metastatic disease with laser interstitial thermal therapy. Front Oncol. 2018;8:499. 30430083 10.3389/fonc.2018.00499 PMC6220072

[12] Szatmári T, Lumniczky K, Désaknai S, et al. Detailed characterization of the mouse glioma 261 tumor model for experimental glioblastoma therapy. Cancer Sci. 2006;97(6):546–553. 16734735 10.1111/j.1349-7006.2006.00208.x PMC11159227

[13] Wakimoto H, Mohapatra G, Kanai R, et al. Maintenance of primary tumor phenotype and genotype in glioblastoma stem cells. Neuro Oncol. 2012;14(2):132–144. 22067563 10.1093/neuonc/nor195 PMC3266381

[14] Mao DD, Gujar AD, Mahlokozera T, et al. A CDC20-APC/SOX2 signaling axis regulates human glioblastoma stem-like cells. Cell Rep. 2015;11(11):1809–1821. 26074073 10.1016/j.celrep.2015.05.027 PMC4481182

[15] Gujar AD, Le S, Mao DD, et al. An NAD+-dependent transcriptional program governs self-renewal and radiation resistance in glioblastoma. Proc Natl Acad Sci U S A. 2016;113(51):E8247–E8256. 27930300 10.1073/pnas.1610921114 PMC5187672

[16] Cain MD, Salimi H, Gong Y, et al. Virus entry and replication in the brain precedes blood-brain barrier disruption during intranasal alphavirus infection. J Neuroimmunol. 2017;308:118–130. 28501330 10.1016/j.jneuroim.2017.04.008 PMC5694394

[17] Kovacs Z, Werner B, Rassi A, Sass JO, Martin-Fiori E, Bernasconi M Prolonged survival upon ultrasound-enhanced doxorubicin delivery in two syngenic glioblastoma mouse models. J Control Release. 2014;187:74–82. 24878186 10.1016/j.jconrel.2014.05.033

[18] Tiwari P, Danish S, Madabhushi A Identifying MRI markers associated with early response following laser ablation for neurological disorders: preliminary findings. PLoS One. 2014;9(12):e114293. 25503713 10.1371/journal.pone.0114293 PMC4263602

[19] Elder JB, Huntoon K, Otero J, et al. Histologic findings associated with laser interstitial thermotherapy for glioblastoma multiforme. Diagn Pathol. 2019;14(1):19. 30767775 10.1186/s13000-019-0794-4 PMC6376796

[20] Wakimoto H, Kesari S, Farrell CJ, et al. Human glioblastoma-derived cancer stem cells: establishment of invasive glioma models and treatment with oncolytic herpes simplex virus vectors. Cancer Res. 2009;69(8):3472–3481. 19351838 10.1158/0008-5472.CAN-08-3886 PMC2785462

[21] Greene C, Campbell M Tight junction modulation of the blood brain barrier: CNS delivery of small molecules. Tissue Barriers. 2016;4(1):e1138017. 27141420 10.1080/21688370.2015.1138017 PMC4836485

[22] Nitta T, Hata M, Gotoh S, et al. Size-selective loosening of the blood-brain barrier in claudin-5-deficient mice. J Cell Biol. 2003;161(3):653–660. 12743111 10.1083/jcb.200302070 PMC2172943

[23] Knowland D, Arac A, Sekiguchi KJ, et al. Stepwise recruitment of transcellular and paracellular pathways underlies blood-brain barrier breakdown in stroke. Neuron. 2014;82(3):603–617. 24746419 10.1016/j.neuron.2014.03.003 PMC4016169

[24] Ayloo S, Gu C Transcytosis at the blood-brain barrier. Curr Opin Neurobiol. 2019;57:32–38. 30708291 10.1016/j.conb.2018.12.014 PMC6629499

[25] Ben-Zvi A, Lacoste B, Kur E, et al. Mfsd2a is critical for the formation and function of the blood-brain barrier. Nature. 2014;509(7501): 507–511. 24828040 10.1038/nature13324 PMC4134871

[26] Stan AC, Casares S, Radu D, Walter GF, Brumeanu TD Doxorubicin-induced cell death in highly invasive human gliomas. Anticancer Res. 1999;19(2A):941–950. 10368637

[27] Ohnishi T, Tamai I, Sakanaka K, et al. In vivo and in vitro evidence for ATP-dependency of P-glycoprotein-mediated efflux of doxorubicin at the blood-brain barrier. Biochem Pharmacol. 1995;49(10): 1541–1544. 7763297 10.1016/0006-2952(95)00082-b

[28] Schober R, Bettag M, Sabel M, Ulrich F, Hessel S Fine structure of zonal changes in experimental Nd:YAG laser-induced interstitial hyperthermia. Lasers Surg Med. 1993;13(2):234–241. 8464310 10.1002/lsm.1900130212

[29] Schulze PC, Kahn T, Harth T, Schwurzmaier HJ, Schober R, Schulze CP Correlation of neuropathologic findings and phase-based MRI temperature maps in experimental laser-induced interstitial thermotherapy. J Magn Reson Imaging. 1998;8(1):115–120. 9500270 10.1002/jmri.1880080123

[30] Schulze PC, Adams V, Busert C, Bettag M, Kahn T, Schober R Effects of laser-induced thermotherapy (LITT) on proliferation and apoptosis of glioma cells in rat brain transplantation tumors. Lasers Surg Med. 2002;30(3):227–232. 11891743 10.1002/lsm.10019

[31] Schulze PC, Vitzthum HE, Goldammer A, Schneider JP, Schober R Laser-induced thermotherapy of neoplastic lesions in the brain—underlying tissue alterations, MRI-monitoring and clinical applicability. Acta Neurochir (Wien). 2004;146(8):803–812. 15254802 10.1007/s00701-004-0293-5

[32] Weidle UH, Niewöhner J, Tiefenthaler G The blood-brain barrier challenge for the treatment of brain cancer, secondary brain metastases, and neurological diseases. Cancer Genomics Proteomics. 2015;12(4):167–177. 26136217

[33] Gan HK, van den Bent M, Lassman AB, Reardon DA, Scott AM Antibody-drug conjugates in glioblastoma therapy: the right drugs to the right cells. Nat Rev Clin Oncol. 2017;14(11):695–707. 28675164 10.1038/nrclinonc.2017.95

[34] Sharma HS, Hoopes PJ Hyperthermia induced pathophysiology of the central nervous system. Int J Hyperthermia. 2003;19(3):325–354. 12745974 10.1080/0265673021000054621

[35] Bastiani M, Parton RG Caveolae at a glance. J Cell Sci. 2010;123(Pt 22):3831–3836. 21048159 10.1242/jcs.070102

[36] Xiao G, Gan LS Receptor-mediated endocytosis and brain delivery of therapeutic biologics. Int J Cell Biol. 2013;2013:703545. 23840214 10.1155/2013/703545 PMC3693099

[37] Mayor S, Parton RG, Donaldson JG Clathrin-independent pathways of endocytosis. Cold Spring Harb Perspect Biol. 2014;6(6). 10.1101/cshperspect.a016758 PMC4031960 24890511

[38] Andreone BJ, Chow BW, Tata A, et al. Blood-brain barrier permeability is regulated by lipid transport-dependent suppression of caveolae-mediated transcytosis. Neuron. 2017;94(3):581–594.e585. 28416077 10.1016/j.neuron.2017.03.043 PMC5474951

[39] Pan W, Stone KP, Hsuchou H, Manda VK, Zhang Y, Kastin AJ Cytokine signaling modulates blood-brain barrier function. Curr Pharm Des. 2011;17(33):3729–3740. 21834767 10.2174/138161211798220918 PMC3939622

