# Supplementary figures and images for: Therapeutic enhancement of blood–brain and blood–tumor barriers permeability by laser interstitial thermal therapy
Source: Neurooncol Adv. 2020 Jun 30;2(1):vdaa071. doi: 10.1093/noajnl/vdaa071 (PMC7344247; doi:10.1093/noajnl/vdaa071)

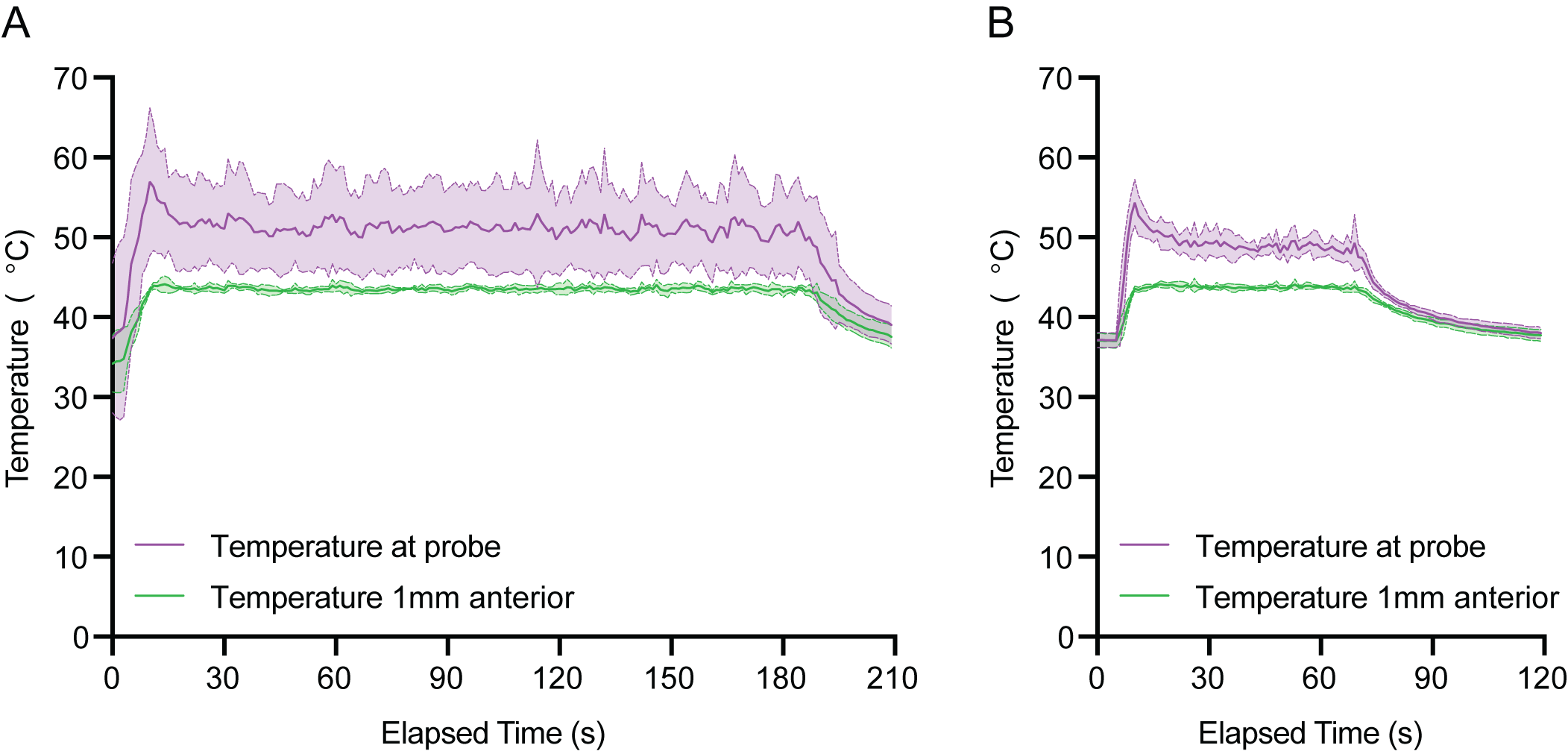

Supplement: vdaa071_suppl_Supplementary_Figure_1 [file vdaa071_suppl_supplementary_figure_1.png]

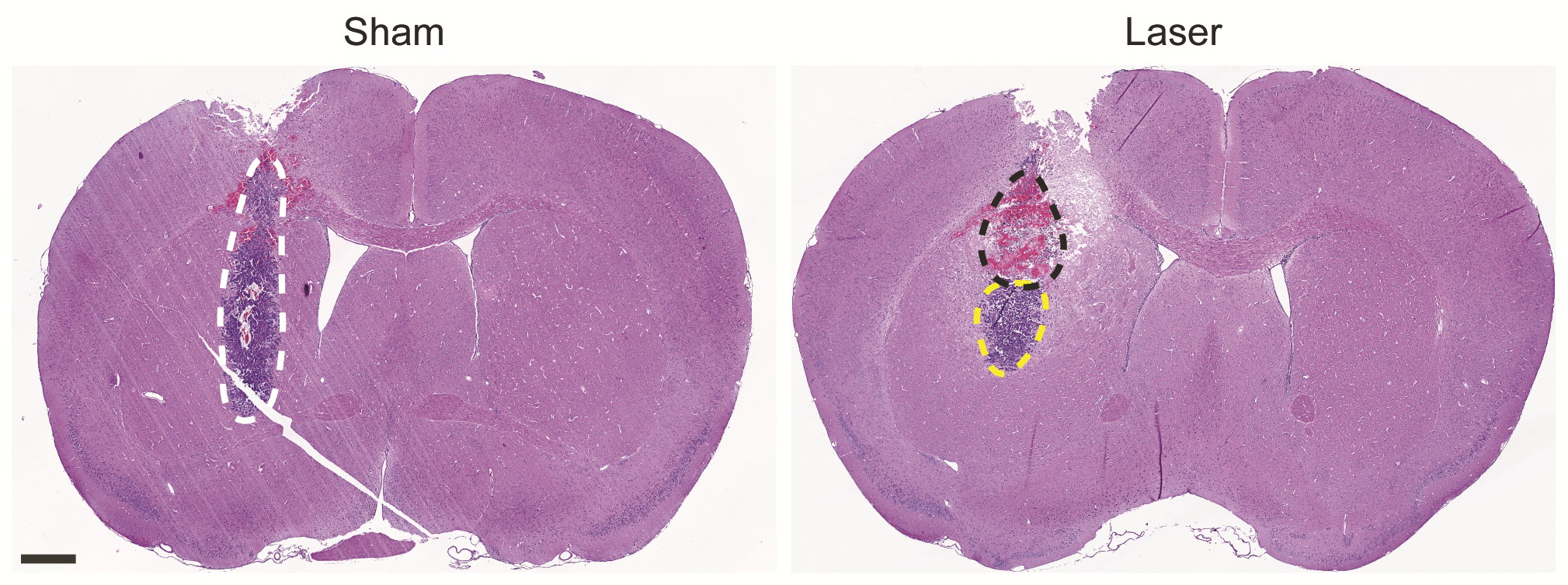

Supplement: vdaa071_suppl_Supplementary_Figure_2 [file vdaa071_suppl_supplementary_figure_2.png]

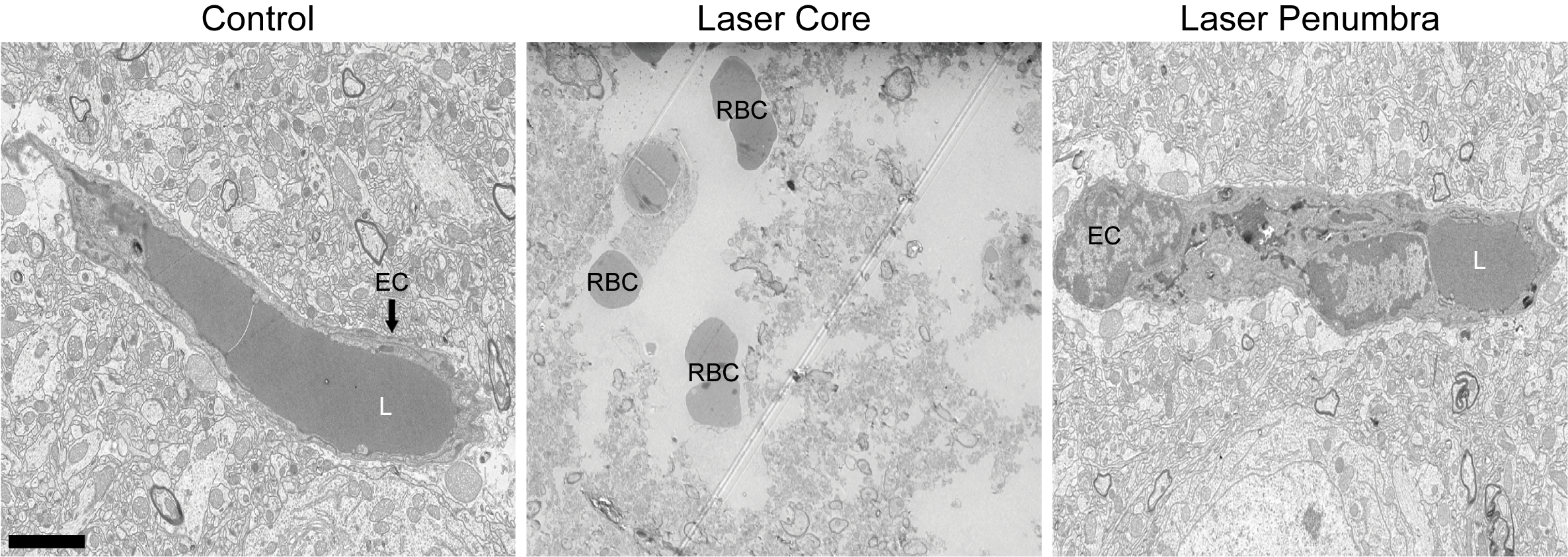

Supplement: vdaa071_suppl_Supplementary_Figure_3 [file vdaa071_suppl_supplementary_figure_3.png]

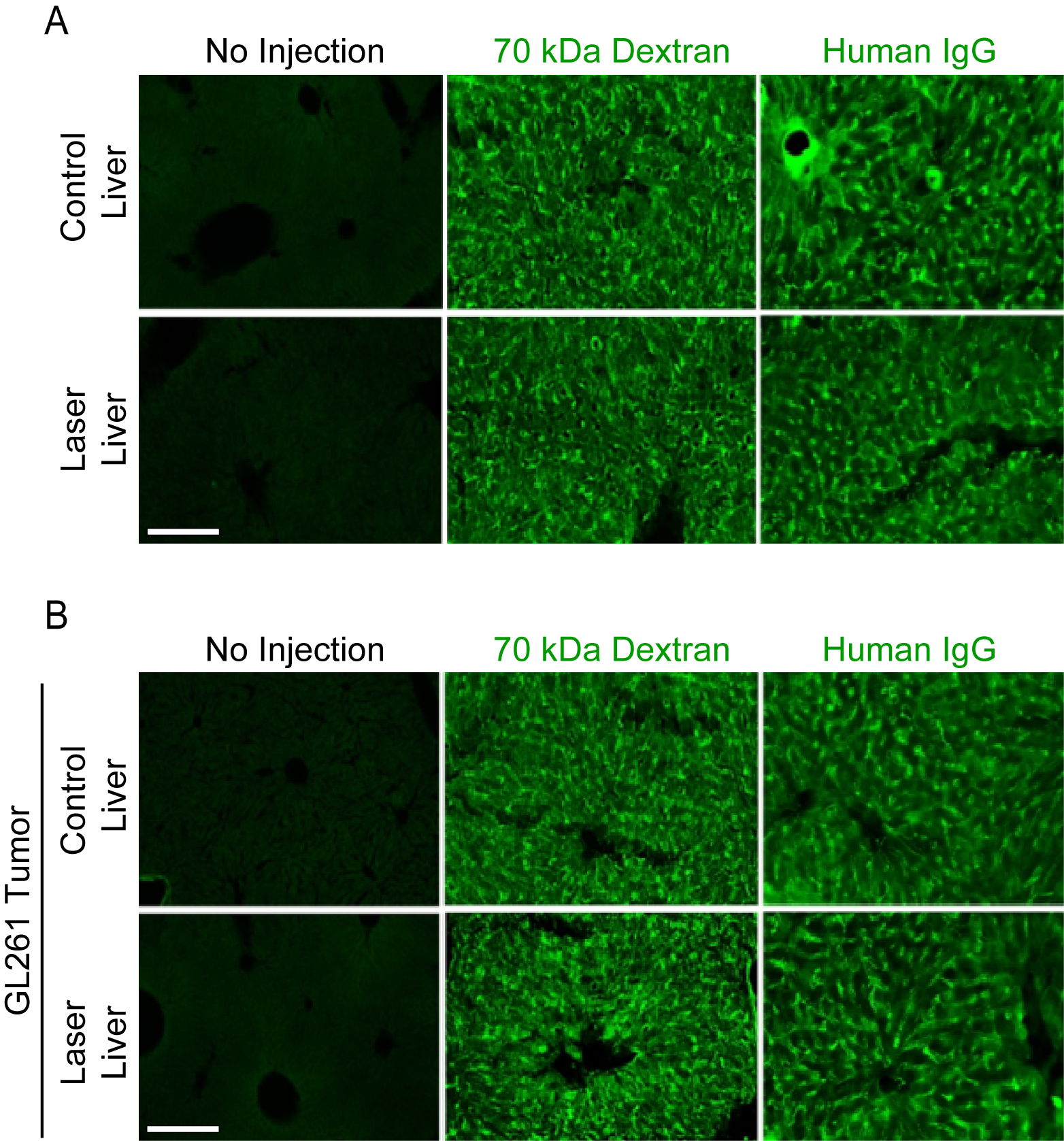

Supplement: vdaa071_suppl_Supplementary_Figure_4 [file vdaa071_suppl_supplementary_figure_4.png]

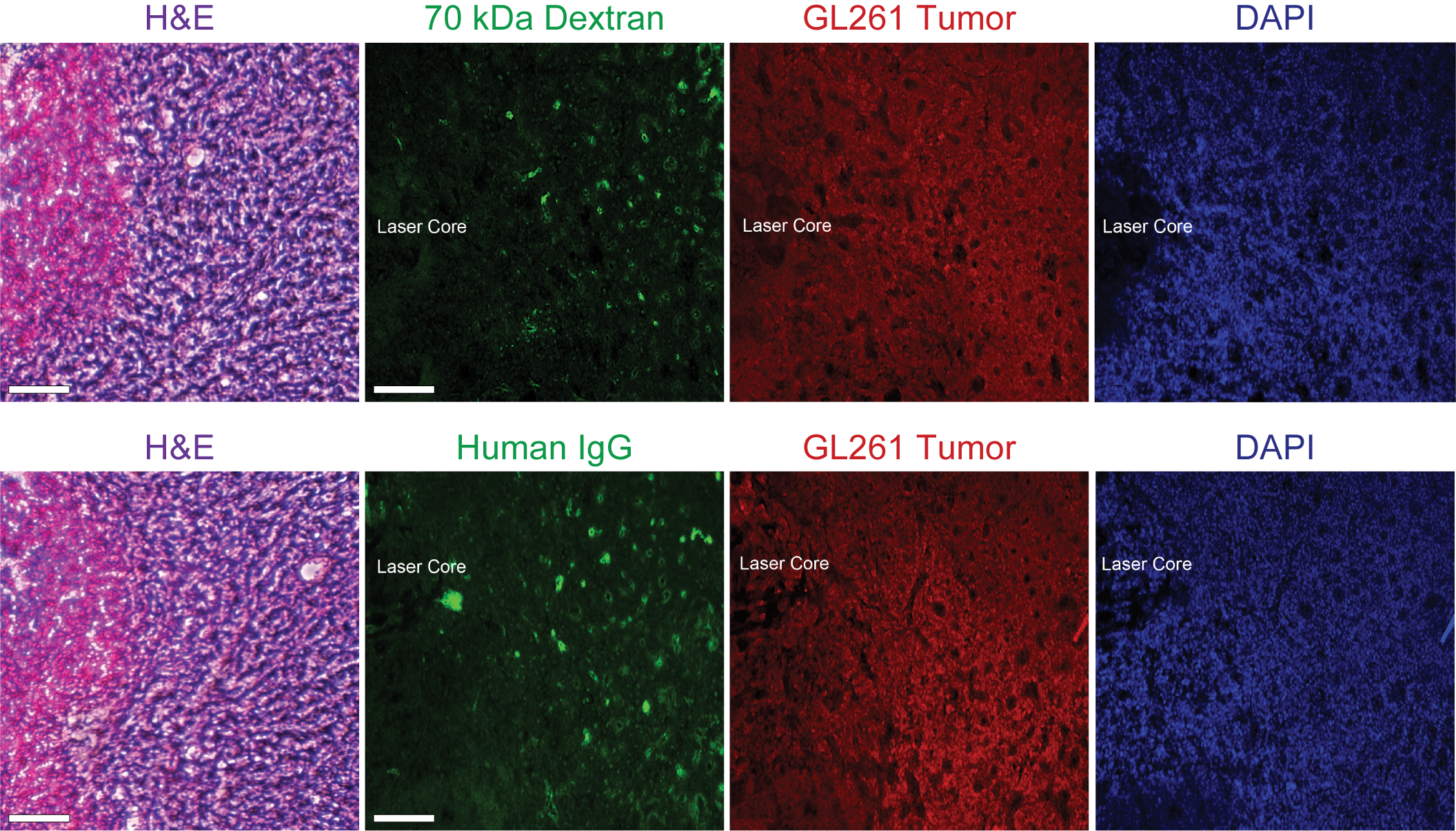

Supplement: vdaa071_suppl_Supplementary_Figure_5 [file vdaa071_suppl_supplementary_figure_5.png]

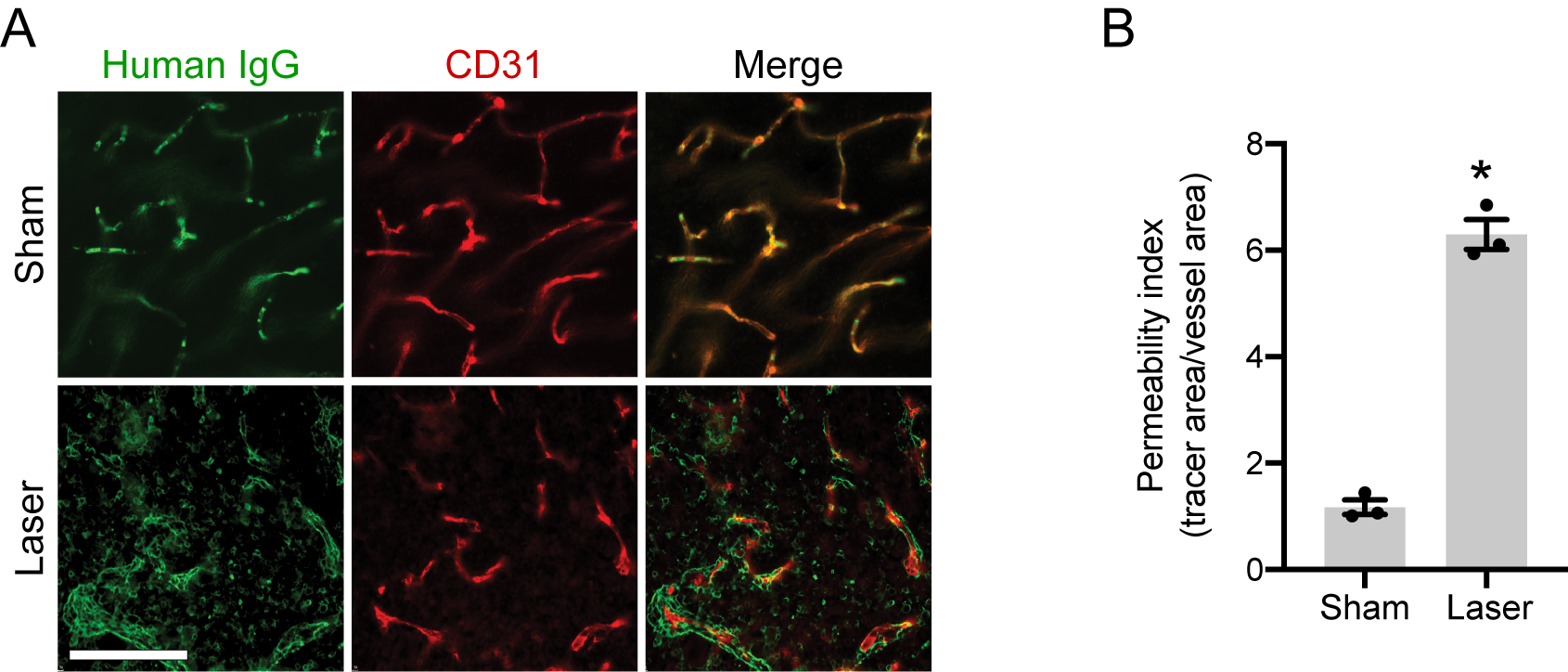

Supplement: vdaa071_suppl_Supplementary_Figure_6 [file vdaa071_suppl_supplementary_figure_6.png]
