# Supplementary material for: Therapeutic enhancement of blood–brain and blood–tumor barriers permeability by laser interstitial thermal therapy
Source: Neurooncol Adv. 2020 Jun 30;2(1):vdaa071. doi: 10.1093/noajnl/vdaa071 (PMC7344247; doi:10.1093/noajnl/vdaa071)
Supplement: vdaa071_suppl_Supplementary_Figure_Legends [file vdaa071_suppl_supplementary_figure_legends.docx]

**Supplementary Figures**

**Figure S1. LITT temperature readings**

**(a)** Temperature recording of laser therapy in tumor-naïve mouse brains for 3 min. **(a)** Temperature recording of laser therapy in tumor bearing mouse brains for 60 seconds. Tumor-bearing mice underwent orthotopic implantation of GL261 7 days prior to laser treatment. Data shown in each panel represents temperature data from 5 mice (solid line mean, shaded area standard deviation) obtained from simultaneous, continuous temperature readings (1 Hz) from one thermo-couple 1 mm rostral to the laser fiber entry site (green) and another thermo-couple immediately adjacent to the laser fiber (purple). After initiation of LITT, brain temperature at the rostral thermocouple is maintained at approximately 43°C for the treatment duration.

**Figure S2. Histology pre- and post-LITT**

Tumor-bearing C57BL/6J mice underwent sham or laser treatment for 60 seconds 10 days after implantation of GL261 cells. On post-treatment day 1, mice were euthanized, and their brains harvested. Specimens were embedded in paraffin and whole mount H&E histology was performed. Compared to sham, laser treatment creates a central necrotic core (black dashed line) surrounded by an area of remaining tumor (yellow dashed line) and edema. Tumor in sham treatment is outlined with white dashed line. Scale bar = 500µm. Representative images are shown (n=3 for each condition).

**Figure S3. Transmission electron microscopy (TEM) of the laser core and penumbra**

C57BL/6J mice were subjected to LITT treatment and brains harvested on post-LITT day 3 for TEM. Representative images of the laser core and penumbra are shown (n=3 for each condition). L = blood vessel lumen; EC = endothelial cell; RBC = red blood cell. scale bar = 2µm.

**Figure S4. Liver analysis confirms successful IV injection of large molecular size tracers**

**(a)** Animals treated with laser to brain (or sham treatment) were injected IV with 70 kDa Dextran or human IgG on post-laser day 3, and livers harvested and processed for immunofluorescence (IF) to assess tracer penetration. Representative images are shown (n=3 for each condition). Scale bar = 100 μm. **(b)** GL261 cells were implanted in mouse brain, and tumor-bearing animals underwent sham (Control) or laser treatment to the brain tumor. Animals were injected IV with 70 kDa Dextran or human IgG on post-laser day 3. Livers were processed as in (a) for IF. Representative images are shown (n=3 for each condition).

**Figure S5. The laser penumbra demonstrates increased BTB permeability following LITT**

RFP-expressing GL261 cells were implanted intracranially and allowed to grow for 10 days. Tumor-bearing mice were treated with LITT to the tumor and then injected intravenously with 70kDa dextran or human IgG on post-LITT day 3. Brains were processed, and adjacent sections subjected to H&E staining, immunofluorescence for tracer penetration and tumor cell visualization (anti-RFP), and DAPI for nuclei. Representative images are shown (n=3 for each condition). The necrotic laser core exhibits substantially less tracer permeability than the surrounding penumbra. Scale bar = 100μsm.

**Figure S6. LITT increases BTB permeability to immunoglobulin**

**(a)** Tumor-bearing animals were treated with LITT 10 days after tumor implantation, injected with intravenous human IgG, and processed for immunofluorescence as indicated. Intravenous human IgG extravasates out of tumor blood vessels into the tumor on post-LITT day 3 whereas IgG is confined to the vasculature in sham-treated tumor. Representative images are shown (n=3 for each condition). CD31 = endothelial cell marker. Scale bar = 300µm **(b)** The tumor permeability index of human IgG was quantified by dividing the area of tracer by area of vessels (µm^2^). Data represent mean+/-SEM. LITT significantly increased IgG permeability index compared to sham (n=3 for each condition, t-test, **P*<0.001).
