# Supplementary material for: Therapeutic enhancement of blood–brain and blood–tumor barriers permeability by laser interstitial thermal therapy
Source: Neurooncol Adv. 2020 Jun 30;2(1):vdaa071. doi: 10.1093/noajnl/vdaa071 (PMC7344247; doi:10.1093/noajnl/vdaa071)
Supplement: vdaa071_suppl_Supplementary_Material [file vdaa071_suppl_supplementary_material.docx]

**Supplementary Materials and Methods**

*Lentivirus*

Viral transduction was performed as described.^1^ A lentiviral expression vector (pGreenfire-CMV-GFP-T2A-Luciferase) was combined with a packaging plasmid (psPAX2) and an envelope plasmid (pCMV-VSV-G) at a ratio of 9:1:8 in Opti-MEM media (Life Technologies). Polyethyleneimine (PEI; Polysciences #24765-2) was added to the plasmid mixture and complexes were allowed to form for 15 minutes at room temperature. The DNA-PEI complexes were added to plated 293LE cells and incubated for 16 hours at 37°C after which media was replaced. 96 hours post-transfection, medium was collected and centrifuged at 1,200xg for 5 minutes at 4°C. The supernatant was filtered through a 0.45-μm filter, followed by addition of Lenti-X concentrator and incubation at 4°C for 6 hrs. Lentiviral particles were subsequently collected by centrifugation at 1,500xg for 45 mins at 4°C, resuspended in cold 1X PBS, and stored at -80°C. For transduction, GL261 cells were plated at a density of 4 x 104 cells/well in a 24-well plate. One day after plating, media was replaced with media containing 4 μg/ml polybrene. Lentiviral particles were added and cells were incubated for 6 hours at 37°C after which media was replaced.

*Magnetic resonance imaging (MRI)*

Mice were imaged with a 4.7-T Agilent DirectDriveTM small-animal MRI system (Agilent Technologies, Santa Clara, CA) equipped with a Magnex/Agilent HD imaging gradient coil (Magnex/Agilent, Oxford, UK) capable of pulsed-gradient strengths of up to 58 G/cm and a gradient rise time ≤ 295 µs. An actively-decoupled 1.7 cm receive-only surface coil was positioned on top of the mouse head. The animal holder assembly was placed inside an 8 cm actively-decoupled volume transmit coil. The body temperature of mice was maintained at 37°C with a warm water pad. T2-weighted (T2W) images in axial view covering the whole mouse brain using a spin-echo sequence were acquired with the following acquisition parameters: TR 2000 ms, TE 45 ms, FOV 20×20 mm2, matrix size 128x128, in-plane resolution 156 μm2, slice thickness 0.8 mm, total scan time 272 s.

*In vivo blood-brain and blood-tumor barrier permeability assays*

Fluorescein permeability assay was performed as described.^2^ At various time intervals post-laser treatment, mice were injected with intraperitoneal dose of sodium fluorescein solution (Sigma-Aldrich, 350 mg/kg, 100 mg/ml in sterile PBS). After 30-45 minutes, mice were anesthetized, and blood was collected by right heart needle aspiration followed by cardiac perfusion with 30cc of PBS and 30cc of 4% paraformaldehyde (PFA) at a rate of 5cc/min to avoid injury to microvessels. Fluorescein concentration was determined by obtaining tissue homogenates that were incubated overnight at 4°C after dilution 1:1 with 2% trichloroacetic acid to precipitate protein. Protein precipitates were then pelleted by centrifugation at 4°C at 3000rpm. Supernatants were diluted 1:1 in borate buffer, pH 11. Sample fluorescence was measured (ex. 480nm; em. 538nm) by Synergy H1 microplate fluorometer (BioTek Instruments, Inc.) and normalized to standard curves of fluorescein concentration, followed by brain tissue weight and then plasma fluorescein concentration to control for fluorescein bioavailability.

10kDa and 70kDa Dextran (Thermo-Fischer Scientific, 200 uL, 18.75 mg/mL in sterile water) or human IgG (Sigma-Aldrich, 200 uL, 8.85 mg/mL in sterile PBS) were injected intravenously retro-orbitally into mice following laser or control treatments. One hour post-injection, mice were anesthetized with 2,2,2 tribromoethanol and perfused with PBS and 4% PFA (Sigma-Aldrich). Brains were harvested and processed for immunohistochemistry with anti-Streptavidin 1:500 (Thermo-Fisher Scientific, Alexa Fluor 594 and 488) as described below.

*Histopathology and immunofluorescence*

Mice were anesthetized with 2,2,2 tribromoethanol and perfused with PBS and 4% PFA (Sigma-Aldrich). Brains were harvested, fixed in 4% PFA overnight, cryopreserved in 30% sucrose, and mounted in OCT (Sigma-Aldrich). For histological studies, brain sections (50 µm) were stained with hematoxylin and eosin. For immunofluorescence, frozen sections (15-50 µm) were blocked in 10% goat serum/5% BSA/PBS/0.5% Triton X-100 and incubated overnight at 4°C with the following primary antibodies: CD31 (1:300, Thermo Fisher Scientific #MA5-13188), anti-human IgG (1:200, Sigma-Aldrich #I6760), anti-RFP (1:200, Thermo Fisher Scientific #MA5-15257), and anti-Claudin V (1:100, Thermo Fisher Scientific #352588; RRID:AB_2532189), followed by incubation with corresponding Alexa fluorophore-conjugated secondary antibodies (1:500, Thermo-Fisher Scientific). Sections were mounted on glass slides with Fluoroshield DAPI (Sigma-Aldrich).

For *in vitro* immunofluorescence, bEnd.3 cells (ATCC CRL-2299) were plated in 96-well plates and grown to confluence. Cells were then incubated at 43°C for one hour and then at 37°C for 24 hours. Control and heated cells were then fixed on ice with 100% methanol for 2 minutes, blocked at room temperature for one hour with blocking solution as described above, and then incubated with anti-Claudin V antibody (1:100, Thermo-Fisher Scientific #35-2500) for 2 hours at room temperature. Cells were then incubated in corresponding Alexa fluorophore-conjugated secondary antibody (1:500, Thermo-Fisher Scientific) for one hour and Hoechst (1:5000, Thermo-Fisher Scientific 62249) for 5 minutes.

Fluorescent images were taken using an automated inverted microscope (Leica Microsystems). Analysis of immunofluorescence images was performed via corrected total fluorescence on FIJI.

*Doxorubicin permeability and quantification*

For quantification, doxorubicin (Sigma-Aldrich, 16 mg/kg) dissolved in sterile water was injected intravenously retro-orbitally into mice. Each mouse was anesthetized and perfused by transcardiac method for 30 minutes with PBS to flush unabsorbed doxorubicin from cerebral vessels. The sites of laser treatment and/or sham treatment control were harvested. Brain homogenates were generated by homogenization (Bead Blaster 24, Benchmark Scientific) and refrigeration for 24 hours in 20 vols of acidified ethanol at 4°C. Tissues were centrifuged at 16,000g for 25 minutes at 4 C, and the supernatant was stored at −20°C.^3^ The concentration of doxorubicin present was measured by fluorimeter (Cytation 5, BioTek, USA; excitation: 480 nm; emission: 590 nm), and the value determined by taking the average of at least three fluorometric readings. For BLI and survival experiments, 12 mg/kg of doxorubicin was used.

*Transmission electron microscopy (TEM)*

Horseradish peroxidase (HRP) type II (Sigma-Aldrich, #P8250-50KU, 0.5 mg/g body weight dissolved in 0.4 ml PBS) was injected into the retro-orbital vein of 5 to 6-week-old female C57BL/6J mice. After 30 minutes, brains were harvested and fixed by drop immersion, first in 5% glutaraldehyde/4% PFA/0.1M sodium-cacodylate for 1 hour at room temperature, then overnight at 4°C in 4% PFA/0.1M sodium-cacodylate. Following fixation, brains were washed overnight in 0.1M sodium-cacodylate. Coronal vibratome free-floating sections of 50 μm were collected, post-fixed in 1% osmium tetroxide and 1.5% potassium ferrocyanide, dehydrated, and embedded in epoxy resin. Ultrathin sections (80 nm) were then cut from the block surface, collected on copper grids, and counterstained with Reynold’s lead citrate. An Olympus VS120-SL 5 slide scanner was used to image adult HRP tracer permeability assays. A 1200EX electron microscope (JOEL) equipped with a 2k CCD digital camera (AMT) was used for all TEM studies. Images were analyzed using ImageJ (NIH).

**References**

1. Mao DD, Gujar AD, Mahlokozera T, Chen I, Pan Y, Luo J, et al. A cdc20-apc/sox2 signaling axis regulates human glioblastoma stem-like cells. *Cell Rep*. 2015;11:1809-1821

2. Cain MD, Salimi H, Gong Y, Yang L, Hamilton SL, Heffernan JR, et al. Virus entry and replication in the brain precedes blood-brain barrier disruption during intranasal alphavirus infection. *J Neuroimmunol*. 2017;308:118-130

3. Yang FY, Teng MC, Lu M, Liang HF, Lee YR, Yen CC, et al. Treating glioblastoma multiforme with selective high-dose liposomal doxorubicin chemotherapy induced by repeated focused ultrasound. *Int J Nanomedicine*. 2012;7:965-974
